# Testicular ischemia secondary to acute epididymitis: A case report

**DOI:** 10.1097/MD.0000000000033843

**Published:** 2023-05-17

**Authors:** Zhichao Wang, Mengzhen Qiu, Xinghua Gao, Longyang Zhang

**Affiliations:** a Department of Urology, Jinan Central Hospital, Shandong University, Jinan, Shandong Province, China; b Department of Urology, Central Hospital Affiliated to Shandong First Medical University, Jinan, Shandong Province, China.

**Keywords:** acute epididymitis, complications, testicular ischemia

## Abstract

Rare side effects of acute epididymitis include testicular infarction and ischemia. Distinguishing them from testicular torsion is challenging, both clinically and radiologically. However, only a few such cases have been reported to date.

**Patient concerns:**

A 12-year-old child presented with persistent right testicular pain for 3 days. It developed after trauma and was accompanied by gradual swelling and enlargement of the right scrotum, with nausea and vomiting. Scrotal color Doppler ultrasonography demonstrated right epididymitis, right scrotal wall swelling, and right testicular torsion. Routine blood tests revealed leukocyte and neutrophil counts were both above normal.

**Diagnosis:**

Scrotal exploration revealed edema and adhesions in all layers of the scrotal wall. The right testicle was pale. The patient was diagnosed with testicular ischemia secondary to acute epididymitis.

**Interventions:**

The patient underwent simultaneous lower spermatic cord sheath dissection and decompression, testicular sheath reversal, and right testicular fixation.

**Outcomes:**

Blood flow to the testicles gradually recovered after decompression, as did the color. Postoperatively, the patient’s scrotal swelling and pain improved significantly.

**Lessons:**

Despite the rarity of this condition, it is a potentially serious consequence of epididymitis and should be considered when patients experience sudden scrotal pain.

## 1. Introduction

Acute epididymitis is a common clinical condition of the urinary tract. Clinical manifestations include pain and swelling of one testicle or epididymis, accompanied by fever and urinary tract irritation. Anti-infection therapy remains the primary treatment strategy. Severe infections of the epididymis and testes can lead to secondary ischemia, which is a serious complication. However, secondary ischemia of the testes has rarely been reported, as a complication of epididymitis.^[1,2]^ Herein, we have reported a case of testicular ischemia secondary to acute epididymitis.

## 2. Case report

**Figure 1.**
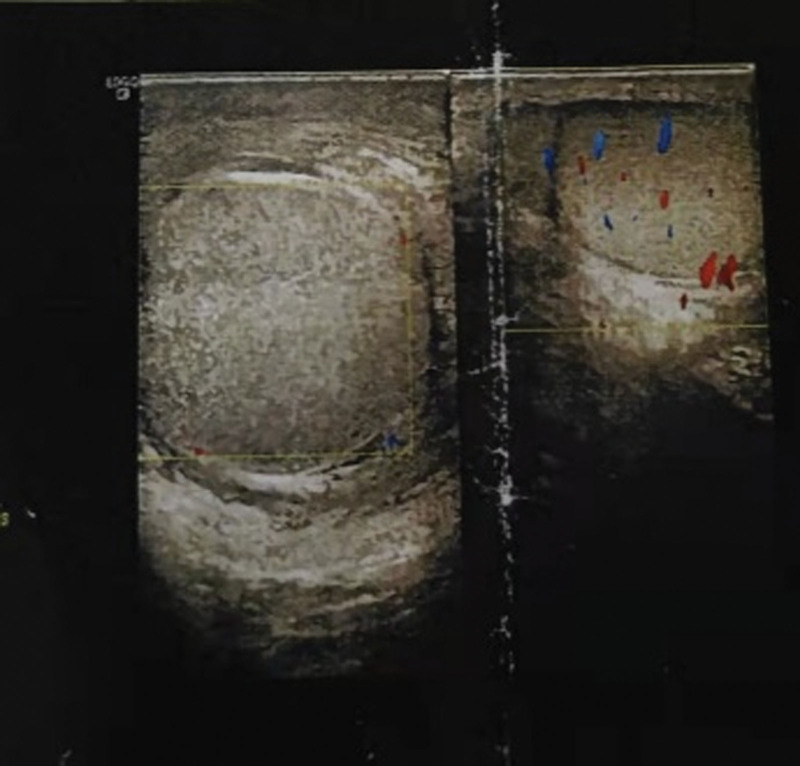
Doppler ultrasonography reveals right epididymitis, right scrotal wall swelling, and right testicular torsion.

**Figure 2.**
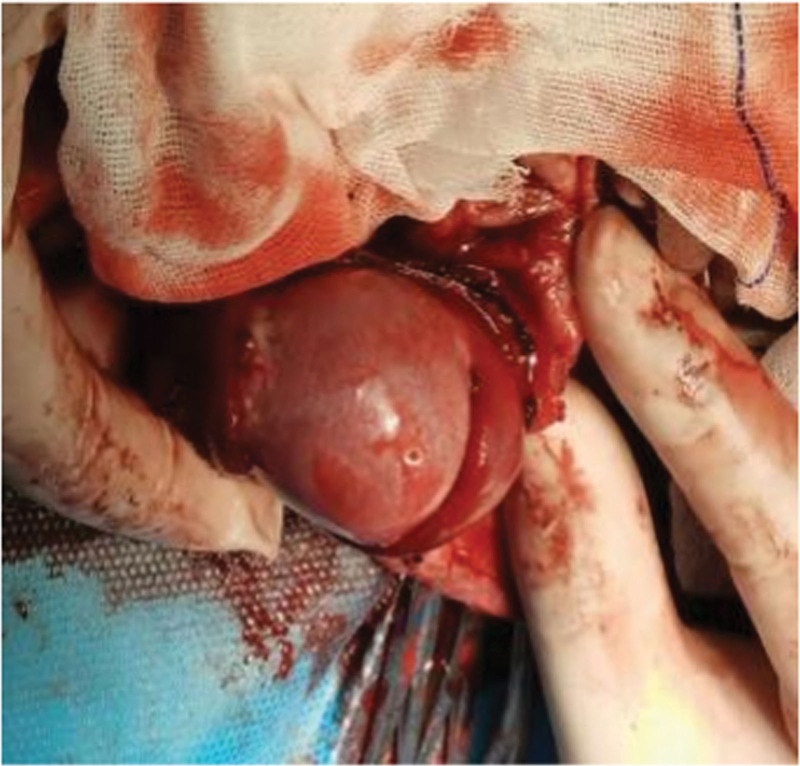
Improvement in blood flow and color of the testis after decompression.

The patient, a 12-year-old child was admitted to the hospital with “trauma to the right scrotum 3 days ago.” The patient had persistent pain in the right scrotum for 3 days after the trauma, which gradually increased. It was accompanied by gradual swelling and enlargement of the right scrotum. The patient also experienced nausea and vomiting. Scrotal color Doppler ultrasonography performed one day previously at another hospital had revealed right epididymitis, right scrotal wall swelling, and right testicular torsion (Fig. 1). The patient had been physically fit prior to this admission, and had no significant past medical or surgical history. On physical examination, there was no redness or swelling of the urethra, abnormal discharge, swelling of the skin of the right scrotum, an increase in the volume of the right scrotum, obvious tenderness, indistinct demarcation of the testis and epididymis, and no obvious abnormality of the left testis. The preliminary diagnosis was acute epididymitis with or without testicular ischemia. After admission, routine blood tests demonstrated a leukocyte count of 25.37 × 10^9^/L and a neutrophil count of 0.86. Scrotal color Doppler ultrasonography revealed diffuse lesions in the right testis and epididymis. No significant blood flow signal was observed in the right testis. Emergency scrotal exploration revealed congestion, edema, and adhesions in all layers of the scrotal wall, high tension in the sheath capsule, swelling of the epididymis with obvious congestion, a pale white right testicle, and no torsion of the spermatic cord. Edema and adhesions were observed after the dissection of the lower part of the spermatic cord. Blood flow to the testicles gradually recovered after decompression, and the color became red (Fig. 2). During surgery, lower spermatic cord sheath dissection and decompression, testicular sheath reversal, and right testicular fixation were performed simultaneously. Postoperatively, the tissue (right testicular sheath) sent for pathological examination was observed to be congested and hemorrhagic with some areas of edema. Some areas had more acute and chronic inflammatory cell infiltration in the interstitium. After surgery, the patient’s scrotal swelling and pain improved significantly.

## 3. Discussion

The causes of acute epididymitis include urinary tract infections, prostatitis, and seminal vesiculitis. Scrotal, epididymal, and testicular hematomas may occur after trauma, and they can easily lead to epididymitis. The causative organisms are mostly Escherichia coli, Aspergillus, and Chlamydia. These organisms enter the epididymis through the lumen of the vas deferens. They can also infect the epididymis through the lymphatic system and bloodstream.^[3]^ Without proper treatment, inflammation may spread to the testis within a few days, resulting in epididymo-orchitis.^[4]^ It can occur in all age groups.^[5]^

Antibiotics and analgesics should be administered promptly after the diagnosis of acute epididymitis. The formation of testicular abscess and local ischemia after antibiotic treatment are rare complications of epididymal orchitis.^[6]^ The pathogenesis of testicular ischemia caused by acute epididymal orchitis is unclear as yet. There have been studies in the literature that report that the causes of reduced testicular blood flow may include: the spermatic cord sheath is soft and elastic under physiological conditions, and it has no impact on the testis’s blood flow and lymphatic circulation. Acute epididymitis causes the testicular sheath to enlarge and become edematous. The spermatic cord and associated tissues also swell up and become edematous, compressing the testis and causing it to swell with blood. Due to anatomical factors, the swollen epididymis may compress the testicular arteries and veins. Thickening of the sphincter creates a narrow ring at the time of migration between the spermatic and testicular sheaths, affecting the arterial supply to the testis.^[7]^ Testicular compartment syndrome is defined as an impairment of testicular microcirculation due to increased venous resistance or extraluminal compression, which leads to hypoxia. Once the acute inflammatory process begins within the testis, inflammatory cells start to accumulate. These cells produce large amounts of exudate, leading to peripheral edema that may further compress the blood vessels within the epididymis and testis, resulting in a septal effect within the testis. In addition, extraluminal compression of the testicular microvasculature and elevated venous pressure may lead to septal compartment syndrome.^[8,9]^ In the present case, the patient underwent emergency scrotal exploration due to preoperative ultrasound suspicion of testicular torsion. While intraoperative testicular ischemia was observed, no testicular torsion was reported. The testicular blood flow was gradually restored after decompression. Considering this case in the context of relevant literature, we believe that acute epididymal orchitis can cause secondary testicular ischemia under the influence of multiple factors. Ultrasonography and MRI scans have a higher sensitivity for early testicular secondary ischemia as compared with scrotal color Doppler ultrasonography.^[10]^ Therefore, early ultrasonography and MRI are feasible diagnostic modalities.

When testicular swelling does not decrease after strict conservative treatment, patients with imaging findings demonstrating significantly reduced or absent testicular blood flow should undergo active surgical exploration to improve testicular blood flow, promote inflammation subsidence, prevent testicular ischemia, and preserve the testis.

## Author contributions

**Data curation:** Mengzhen Qiu, Xinghua Gao.

Writing – original draft: Zhichao Wang.Writing – review & editing: Longyang Zhang.
